# Current News

**Published:** 1898-08

**Authors:** 


### Current News.

### NATIONAL DENTAL ASSOCIATION.

The First Annual Meeting of the National Dental Association will be held at Omaha, Neb., commencing at ten A.M., Tuesday, August 30, 1898.

George H. Cushing,
*Recording Secretary.*

N.B.—I wish to state, owing to the conflicting stories in circulation regarding lack of accommodations at Omaha, that I recently visited there to make personal inspection of the hotels, and found the accommodations ample and rates reasonable.

J. N. Crouse,
*Chairman Executive Committee.*

### NATIONAL ASSOCIATION OF DENTAL FACULTIES.

The annual meeting of the National Association of Dental Faculties will be held in the Mercer Hotel, at Omaha, beginning Friday, August 26, at two P.M.

It is to be hoped that all members of the Association will be present at that time.

The Executive Committee will meet on the preceding Thursday at two P.M.

Colleges are notified to present their business at the first session of the committee.

By order of

Jonathan Taft,
*Chairman Executive Committee.*
B. Holly Smith,
*Secretary.*

### HARVARD DENTAL ALUMNI ASSOCIATION.

The Harvard Dental Alumni Association observed its second Alumni Day on Monday, June 27, 1898, at the Dental School building, when nearly one hundred and fifty visitors registered their names with the committee in charge.

The work of the school for each year and class was shown by specimens and patients from all departments, and exhibited distinctive excellence.

The Twenty-seventh Annual Meeting and banquet was held at Young's Hotel, Boston, in the evening, with ninety-three present.

Rev. Edward M. Taylor, D.D., of Cambridge, Mass., the guest of the Association, spoke upon the topic, "The Man for the Twentieth Century," being a most interesting and timely theme. He touched upon the war, and said "it was the noblest that this or any other nation had ever waged."

Professor Eugene W. Smith, dean of the school, spoke of the progress of the school, and that the faculty had recommended the conferring of the degree of *cum laude* on students who spend three full years in the school and pass examinations in distinctive excellence. Professor Thomas Fillebrown referred to the post-graduate course and the entrance examinations.

Harry L. Grant, A.B., responded for the class of 1898.

These officers were elected for the ensuing year: President, Frederick Bradley, '86, Newport, R. I.; Vice-President, Edwin C. Blaisdell, '83, Portsmouth, N. H.; Secretary, Waldo E. Boardman, '86, Boston, Mass.; Treasurer, Harry S. Parsons, '92, Boston, Mass.

*Executive Committee.*—Waldo E. Boardman, '86, *ex officio*, chairman, Boston; William P. Cooke, '81, Boston; Patrick W. Moriarty, '89, Boston.

The Council is composed of the officers of the Association.

Waldo E. Boardman,
*Secretary.*

Boston, July 1, 1898.

